# Effect of Patulin from *Penicillium vulpinum* on the Activity of Glutathione-S-Transferase and Selected Antioxidative Enzymes in Maize

**DOI:** 10.3390/ijerph14070825

**Published:** 2017-07-22

**Authors:** Ahmed A. Ismaiel, Jutta Papenbrock

**Affiliations:** 1 Department of Botany and Microbiology, Faculty of Science, Zagazig University, Zagazig 44519, Egypt; microbiologist_80@yahoo.com; 2 Institut für Botanik, Leibniz Universität Hannover, Herrenhäuser Straße 2, 30419 Hannover, Germany

**Keywords:** glutathione-S-transferase (GST), antioxidants, maize, patulin (PAT), *Penicilliumvulpinum*

## Abstract

The mycotoxin patulin (PAT) was purified from *Penicillium vulpinum* CM1 culture that has been isolated from a soil cultivated with maize. The effect of PAT and of a fungal culture filtrate on the activities of glutathione-S-transferase (GST) and some antioxidant enzymes viz. ascorbate peroxidase (APX), glutathione reductase (GR), dehydroascorbate reductase (DHAR) and monodehydroascorbate reductase (MDHAR) was investigated in roots and shoots of 8-day-old maize seedlings. PAT and culture filtrate caused significant reduction effects in a dose-related manner on the total GST activity. Upon application of the high PAT concentration (25 μg·mL^−1^) and of the concentrated fungal filtrate (100%, *v/v*), the reduction in GST activity of roots was 73.8–76.0% and of shoots was 60–61.7%. Conversely, significant increases in the activities of antioxidant enzymes were induced. Application of 25 μg·PAT·mL^−1^ increased APX, GR, DHAR, and MDHAR activity of root by 2.40-, 2.00-, 1.24-, and 2.16-fold, respectively. In shoots, the enzymatic activity was increased by 1.57-, 1.45-, 1.45-, and 1.61-fold, respectively. Similar induction values of the enzymatic activity were obtained upon application of the concentrated fungal filtrate. This is the first report describing the response of GST and antioxidant enzyme activities of plant cells to PAT toxicity.

## 1. Introduction

**Figure 1:**
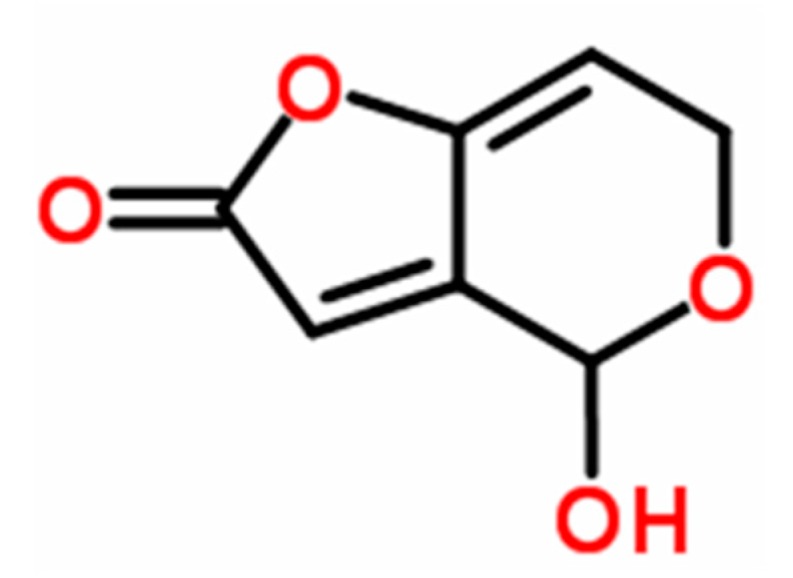
Structural formula of PAT (C_7_H_6_O_4_).

Patulin (4-hydroxy-4*H*-furo[3,2-c] pyran-2(6*H*)-one) (PAT, Figure 1) is a mycotoxin produced by large number of fungal species within several genera such as *Aspergillus*, *Byssochlamys, Eupenicillium*, *Paecilomyces*, and *Penicillium* [1], including *Penicillium vulpinum* (Cooke & Massee) Seifert & Samson (formerly *P. claviforme*), a phytotoxic fungus associated with decaying plant roots [2,3]. This fungal species was reported to produce several mycotoxins such as α-cyclopiazonic acid, griseofulvin, PAT, and roquefortine C, in addition to alkaloids (cyclopeptin, cyclopenin, meleagrin, and oxaline) [4]. Of these mycotoxins, PAT is the most potent mycotoxin and is frequently found in vegetables and fruits (in particular in ripe apples and their juices) [5,6]. PAT is a wide-spectrum biocide and has been reported to be toxic to viruses, bacteria, fungi, protozoa, *Artemia salina* (brine shrimp) larvae, HeLa cells, mammals, and plants [7]. In mammals, its acute toxic effects are primarily related to impairment of kidney functions and induction of stomach ulceration [1]. Besides its ability to cause oxidative damage [8], PAT has been shown to cause genotoxic, immunotoxic, neurotoxic, and teratogenic effects [9,10,11] and possibly possess carcinogenic activity [7]. PAT was also reported to cause other severe health effects in mammals such as convulsions, nausea, ulceration, lung congestion, and epithelial cell degeneration [11], in addition to its effect on reproduction in males at both histopathological and hormonal levels [12]. The phytotoxicity of PAT has been characterized (as reviewed by Ismaiel and Papenbrock [13]), it has been shown to inhibit plant elongation phases due to its effect on root development, cell division, and cell development accompanied with reduction in both seeds and flowers number and seed weight [14,15].

In plants, evolution of aerobic metabolic processes (such as respiration and photosynthesis) inevitably led to the production of reactive oxygen species (ROS) in mitochondria, chloroplasts, and peroxisomes. Excessive generations of ROS were significantly obtained when plant exposed to biotic and abiotic stresses [16]. The ROS such as hydrogen peroxide, superoxide, and organic peroxides are very lethal and disrupt the redox status of the cell resulting in oxidative stress thereby causing extensive damage to protein, carbohydrate, lipid, and nucleic acid content of plant cell [17,18]. The ROS toxicity is counter-balanced by antioxidative system defense of plant which comprises enzymatic as well as non-enzymatic antioxidant machinery [19]. The enzymatic components include superoxide dismutase (SOD), catalase (CAT), ascorbate peroxidase (APX), glutathione-S-transferase (GST), glutathione reductase (GR), monodehydroascorbate reductase (MDHAR) and dehydroascorbate reductase (DHAR). The non-enzymatic components include low molecular compounds like ascorbate (AsA), GSH, carotenoids, and tocopherols. Most of the antioxidative enzymes are electron donors and react with free radicals forming innocuous end products (such as water). During this process, binding of the ROS to active enzyme sites followed by conversion to non-toxic products occurs [19]. Among these enzymes, APX, MDHAR, DHAR, and GR are enzymes of AsA-GSH cycle (also referred to as Halliwell–Asada pathway) which plays a crucial role in combating oxidative stress and involves successive oxidation and reduction of AsA, GSH, and NADPH [20]. Plant GSTs’ activity also protect cells from oxidative stress and xenobiotic and heavy-metal toxicity due to their ability to catalyze the conjugation of GSH to a wide variety of hydrophobic and electrophilic substrates forming less- or non-toxic peptide derivatives [21].

Although PAT displayed several diverse biological activities, its mechanism of cellular toxicity is still a matter of debate [10]. It is well documented that PAT inhibits protein, RNA, and DNA synthesis. Moreover, the genotoxic activity has been suggested based on its ability to conjugate with sulfhydryl groups (–SH) and induce oxidative damage [8]. It was also postulated that PAT causes damage of the human genetic material based on its capacity to induce single and double strand breaks and form DNA–DNA cross-links [10,22]. An interpretation suggested that PAT interact with sodium or proton transport based on its capacity to inhibit Na^+^/K^+^ ATPase activity of plasma membrane [7,10]. It was believed that PAT causes immediate respiratory inhibition in maize seedlings and both germinating apple pollen and soybean suspension cultures [15,23].

Due to the reactivity of PAT with cellular nucleophiles (by covalent binding), in particular with thiol and amino groups of proteins and GSH, the inhibition of various enzymes such as alcohol and lactic dehydrogenases and muscle aldolase was demonstrated [24,25]. However, no studies have been carried out on the effect of PAT on GST and other antioxidant enzymes in plants. Nevertheless, only two studies in the literature have focused on the inhibition of GST activity after incubating primary liver cells with PAT [26,27] and this inhibitory effect may be assigned to the formation of PAT/GSH adducts. Thus, assessing a correlation between PAT phytotoxicity and activities of antioxidant enzymes becomes essential. We have already described the phytotoxic activity of PAT via its effect on the endogenous GSH of maize [15]. In this paper, we determined the effects of PAT on antioxidant enzymes of maize seedlings in order to better characterize the mode of PAT phytotoxic action.

## 2. Materials and Methods

### 2.1. Chemicals

Patulin (PAT), reduced glutathione (GSH), oxidized glutathione (GSSG) 1-chloro-2,4-dinitrobenzene (CDNB), dithiothreitol (DTT), polyvinylpyrrolidone (PVP), ethylenediamine tetraacetic acid (EDTA) disodium salt, 5,5’-dithiobis (2-nitrobenzoic acid) (DTNB), potassium phosphate, phenylhydrazine hydrochloride, Tris-HCl, L-ascorbic acid (AsA), L-dehydroascorbate, ascorbate oxidase (from *Cucurbita* spp.), H_2_O_2_, NADH, and bovine serum albumin (BSA) were obtained from Sigma-Aldrich (Taufkirchen, Germany). All other chemicals and solvents used were of highest purity commercially available.

### 2.2. Culture Conditions

*Penicillium vulpinum* CM1, a local soil isolate recovered from a soil sample cultivated with maize, was identified and selected based on its capability of producing the mycotoxin PAT from our preliminary studies [15]. Fungal culture was stored at 7 °C after subculturing on yeast–sucrose agar (YES; yeast extract 20 g, sucrose 200 g, agar 20 g, distilled water 1 L) which was repeated in 3- to 5-month intervals.

Using a hemocytometer, the fungal spore concentration was adjusted to 2 × 10^6^ mL^−1^ after harvesting the spores from 5-day-old cultures by flooding the slants with 0.1% Tween 20 and then scrapping off the spores. One milliliter of freshly prepared spore suspension was inoculated into a 500 mL Erlenmeyer flask containing 100 mL YES broth (pH 6.0), after which fermentation was carried out statically in the dark at 30 °C for 10 days.

### 2.3. PAT Extraction and Quantification

In order to extract PAT from the culture flasks of *P. vulpinum*, the cultures were filtered through Whatman no.1. filter papers. Using 2 N HCl, the fungal filtrate was adjusted to pH 2.0 and was then defatted with *n*-hexane. Using a separating funnel, PAT was extracted from the filtrate by shaking with an equal volume of ethyl acetate for 30 min. The crude extract was filtered over anhydrous sodium sulfate and evaporated under vacuum until dryness using a rotary evaporator (IKA, RV10, Staufen, Germany) [15]. The dried extract was dissolved in ethyl acetate and passed through column chromatography packed with C18 silica gel; thereafter PAT was eluted with ethyl acetate: toluene (30:70, *v:v*) [28]. The resulting fractions were evaporated to dryness and checked for PAT using thin layer chromatography (TLC) on silica gel F-254 plates (20 × 20 cm, 0.25 mm thickness) (Merck, Darmstadt, Germany). The concentrated fractions were dissolved in absolute ethanol, loaded with a reference standard of PAT, and migrated in toluene: ethyl acetate: formic acid (6:3:1, *v:v:v*) as a developing system. PAT appears as yellow spots (R_f_ = 0.52) upon treating the TLC with phenylhydrazine hydrochloride solution (2.0%, *w/v*) followed by heating for 15 min at 130 °C. The yellow spots were scrapped off, eluted with *n*-butanol and their color intensity was monitored at 540 nm [29]. PAT concentration was then obtained from a standard curve.

### 2.4. Preparation of PAT and Fungal Culture Filtrate Concentrations

PAT purified from the chromatographic analyses was pooled and dissolved in chloroform to give a concentration of 10 mg·mL^−1^ which was kept at −20 °C. In the experiments of treating maize seeds with PAT, chloroform was evaporated and the crystalline PAT was dissolved in sterile water to get the desired concentrations of PAT solution at 5, 10, 15, 20, and 25 μg·mL^−1^ (as described later).

Cultures of *P. vulpinum* CM1 grown in YES broth for 10 days at 30 °C (as described earlier) were filtered using a sterile Whatman no.1. filter paper and the resulting filtrate was centrifuged at 4000× *g* for 10 min to get rid of minute debris of fungal biomass and then filter-sterilized using a 0.45 μm cellulose membrane. This sterilized solution was designated as the original (100%) and in order to prepare further desired dilutions at 30%, 60%, and 90%, certain amounts of sterilized distilled water were added to the original solution.

### 2.5. Plant Treatment

Seeds of maize (*Zea mays* cv. Montello) were washed with tap water, surface-sterilized with 70% (*v:v*) ethanol with frequent shaking for 5 min, and thoroughly washed with sterile water. The seeds were then soaked for 8 h in sterile water and allowed to germinate based on the method described by Ismaiel and Papenbrock [15], in which paper towels were cut into small strips (23 × 11 cm) and ten uniform seeds were selected and positioned vertically in a middle line of the paper strip which were rolled loosely into several scrolls. The paper stripe edges were then soaked in plastic tubes containing 14 mL of either PAT or filtrate solution and the seeds were allowed to germinate in the circumference scroll after dark incubation at 22 °C for 8 days and the etiolated seedlings were used for analysis. The experimental conditions included the individual treatment of seeds with different concentrations of PAT (varied from 5 to 25 μg·mL^−1^) and fungal filtrate (varied from 30 to 100%, *v:v*). Seeds grown in sterile water served as control.

### 2.6. Enzyme Assays

In all preparations for analyzing antioxidant enzyme activities in crude enzyme extracts, the freshly harvested roots and shoots either from seedlings treated with PAT or fungal filtrate were ground to fine powder in liquid nitrogen using a pre-chilled mortar and pestle, after which the powder was stored at −80 °C until analysis. The extraction process of all enzymes assayed was performed at 4 °C. For extraction of ascorbate-glutathione recycling enzymes together, 100 mg of the powder sample was extracted into 600 μL of 200 mM potassium phosphate buffer (pH 7.8), 40 mM KCl, 2 mM CaCl_2_, and 1 mM L-AsA (freshly prepared). The homogenate was centrifuged at 14,000× *g* for 20 min at 4 °C and the supernatant was used for the analysis of ascorbate-glutathione recycling enzymes [20]. All enzyme assays were measured using microplate well assays (microtest plate 96-well, Sarstedt AG & Co., Nürnbrecht, Germany) with a final volume of 200 μL per well. Each well in the microplate was read in a microplate spectrophotometer (Synergy Mx Multi-Mode, BioTek, Bad Friedrichshall, Germany) equipped with internal incubator which was programmed at 25 °C with slight shaking for 5 s during determination of the enzymatic reactions.

Following antioxidant enzymes were analyzed: Total GST (EC 2.5.1.18) was extracted from the plant samples using the method adopted by Marcacci et al. [30], in which the powder sample (50 mg) was thawed gently at 4 °C in 300 μL 100 mM potassium phosphate buffer (pH 7.8) containing 5 mM EDTA, 5 mM DTE, and 1% PVP. The homogenate was then centrifuged twice at 20,000× *g* for 15 min at 4 °C and the supernatant is analyzed immediately for enzyme activity. The GST activity was determined spectrophotometrically according to Habig et al. [31] using CDNB as a substrate. In each well, the assay mixture contained 150 μL of 100 mM potassium phosphate buffer (pH 6.5), 10 μL of 20 mM GSH, and 10 μL of 20 mM CDNB and the reaction was initiated by the addition of 30 μL of crude extract. The change in absorbance at 340 nm was monitored for 5 min. Wells containing the reaction mixture without the crude extract served as control. One unit of enzyme activity was defined as the amount of enzyme required to form 1 μmol conjugated product min^−1^ using an extinction coefficient of 9.6 mM^−1^·cm^−1^ at 340 nm.

APX (EC1.11.1.11) was assayed according to the method of Elavarthi and Martin [32]. The APX assay mixture contained 50 mM potassium phosphate buffer (pH 7.0), 0.5 mM H_2_O_2_, 0.5 mM AsA and 10 μL of crude extract. The reaction was initiated by last addition of 5 μL H_2_O_2_ using an 8-channel pipette and the activity was determined from the decrease in absorbance at 290 nm (due to oxidation of AsA) for 3 min. In order to correct the values resulted from non-enzymatic oxidation of AsA, wells containing the reaction mixture without the crude extract were prepared and served as blank. Specific activity was calculated in terms of μmol of AsA per mg of protein per min from the extinction coefficient (2.80 mM^−1^·cm^−1^ for reduced AsA).

GR (EC 1.6.4.2) activity was assayed by monitoring the increase in absorbance at 412 nm resulted from the reduction of 5,5’-dithiobis (2-nitrobenzoic acid) (DTNB) to 2-nitrothiobenzoic acid (TNB) by GSH [32]. In each well, the assay mixture contained 50 mM potassium phosphate buffer (pH 7.0), 0.75 mM DTNB, 0.1 mM NADPH, and 30 μL of crude extract and the reaction was started by addition of 40 μL of 5 mM GSSG. The enzymatic activity rate was determined for 5 min and the specific activity was calculated in terms of μmol TNB per mg of protein per min using 14.15 M^−1^·cm^−1^ extinction coefficient.

DHAR (EC 1.8.5.1) activity was assayed by measuring the GSH-dependent reduction of dehydroascorbate as described by Murshed et al. [20]. The assay mixture contained 50 mM Tris-HCl buffer (pH 7.0), 0.1 mM EDTA, 2.5 mM GSH, 8 mM dehydroascorbate and 15 μL of crude extract. Using an 8-channel pipette, 5 μL freshly prepared dehydroascorbate was added last to initiate the reaction in each well and the increase in absorbance was recorded at 265 nm for 5 min. Wells containing the reaction mixture without the crude extract were served as blank to correct the values resulted from non-enzymatic reduction of dehydroascorbate by GSH. Specific activity was calculated in terms of μmol dehydroascorbate per mg of protein per min using 14.15 mM^−1^·cm^−1^ extinction coefficient.

MDHAR (EC 1.6.5.4) activity was assayed by measuring the decrease in absorbance at 340 nm resulted from the NADH oxidation [20]. In each well, the assay mixture contained 50 mM Tris-HCl buffer (pH 7.6), 2.5 mM AsA, 0.25 mM NADH, 0.5 U of ascorbate oxidase and 15 μL of crude extract. Ascorbate oxidase (from *Cucurbita* spp.) was added last to start the reaction and the enzymatic activity rate was determined for 5 min. Specific activity was calculated in terms of μmol NADH oxidized per mg of protein per min using 6.22 mM^−1^·cm^−1^ extinction coefficient.

The protein content of root and shoot powder samples either treated with PAT or fungal filtrate was determined spectrophotometrically according to Bradford [33] using BSA as a standard.

### 2.7. Data Analysis

Each experiment was carried out three times in triplicate and data were expressed as the mean ± standard deviation (SD). Statistical significance was evaluated using analysis of variance (ANOVA, SPSS software version 22, IBM Corp., New York, NY, USA) test followed by the least significant difference (LSD) test at 0.05 level.

## 3. Results

### 3.1. Effect of PAT and Culture Filtrate of P. vulpinum CM1 on GST Activities

GST activity in both root and shoot tissue of maize was analyzed under the influence of different concentrations of PAT (Table 1). A remarkably reduced activity of GST resulted in both root and shoot following treatment with different PAT concentrations. In particular, the reduction of GST activity in both root and shoot due to treatment with PAT was dose-dependent. At all tried concentrations of PAT, significant reduction (*p* ≤ 0.05) in the enzyme activity of root and shoot was obtained, as compared with control treatment (without PAT). The greater decrease in GST activity was more pronounced in root than in shoot. The total GST activity in root and shoot declined by 47.0% and 37.8%, respectively; following treatment with 5 μg·PAT·mL^−1^. The decline rate in enzyme activity increased to 55.7% and 43.4%, respectively; after treatment with 10 μg·PAT·mL^−1^. Treatment with 25 μg·PAT·mL^−1^ (the higher dose) resulted in 73.8% and 60.1% drop in total GST activity, respectively. A similar trend was evident in the GST activity of root and shoot following treatment with fungal filtrate (Table 2). The reduction in GST activity of root was significant (*p* ≤ 0.05) at all tried concentrations of fungal filtrate when compared with control treatment. Interestingly, the shoot showed insignificant reduction levels of GST activity at different fungal filtrate concentrations (except the concentrated fungal filtrate, 100%). At 100% fungal filtrate, the drop in the total GST activity of root and shoot reached 76.0% and 61.7%, respectively. It is noteworthy to indicate that the GST activity in roots was significantly higher than that of its shoots.

### 3.2. Response of Antioxidant Enzyme Activities

**Figure 2:**
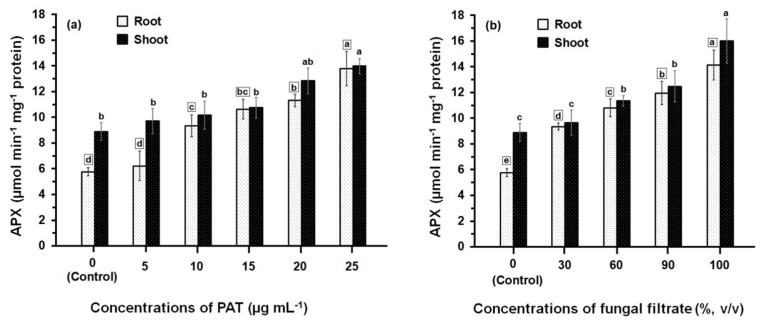
APX activity in root and shoot of 8-day-old maize seedlings treated with different concentrations of PAT (**a**) and fungal filtrate (**b**). In this figure, data are shown as the mean ± SD of triplicate measurements from three independent experiments. Different letters either for roots (in small squares) or shoots (without squares) at different concentrations are statistically different (LSD test, *p* ≤ 0.05).

Data presented in Figure 2 illustrate the APX activity of root and shoot of maize seedling following treatment with PAT (Figure 2a). The APX activity was gradually increased upon increasing the concentrations of PAT. This increase in APX activity was significant (*p* ≤ 0.05) in root tissues treated with PAT concentration starting with 10 μg·mL^−1^ onwards, where as in shoot tissues, the significant increase in APX activity was obtained at the higher PAT concentrations (20 μg·mL^−1^ and 25 μg·mL^−1^). The APX activity measured either in root or shoot treated with 25 μg·mL^−1^ exceeded 2.40- and 1.57-fold more than those measured in their respective controls. Data further showed that APX activity was lower in root than shoot and significant differences (*p* ≤ 0.05) in the APX activities were obtained at all PAT concentrations except at 15 μg·mL^−1^ and 25 μg·mL^−1^. Similarly to the effect of PAT on the APX activity, treating the root and shoot with culture filtrate of *P. vulpinum* showed an increase in the enzyme activity which was dose-related (Figure 2b). Meanwhile, the activity resulting from either treatment of root or shoot with the different concentrations of culture filtrate was relatively higher than those resulting from treatment with PAT concentrations. The concentrated fungal filtrate (100%, *v/v*) stimulated the APX activity in root 2.6-fold and in shoot 1.8-fold, when compared with the respective controls. It was also obvious that the enzyme activity was greater in shoot than in root, and significant differences (*p* ≤ 0.05) were obtained at 100% fungal filtrate.

**Figure 3:**
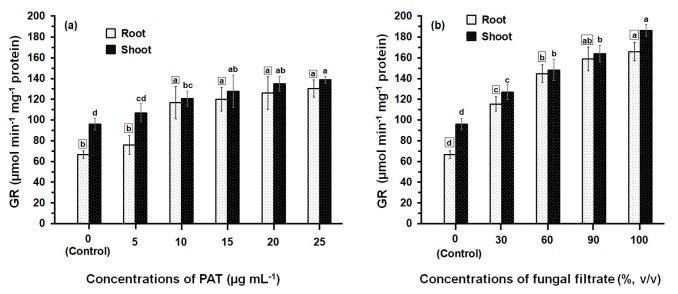
GR activity in root and shoot of 8-day-old maize seedlings treated with different concentrations of PAT (**a**) and fungal filtrate (**b**). Shown are the mean values ± SD of triplicate parallel determinations. All experiments in this figure were performed three times, with similar results. Different letters either for roots (in small squares) or shoots (without squares) at different concentrations are statistically different (LSD test, *p* ≤ 0.05).

The activity of GR determined in both root and shoot tissues of maize was greatly affected by PAT and fungal filtrate treatments (Figure 3). In both cases of application either with PAT or fungal filtrate, the GR was significantly enhanced (*p* ≤ 0.05) in root and shoot tissues, compared with control treatments and this enhancement was concentration-related. Treatments of root with 5 to 25 μg·PAT·mL^−1^ resulted in a 1.14- to 2-fold increase, as compared to control. In shoot, the PAT concentrations resulted in a 1.14- to 1.45-fold increase (Figure 3a). In the case of root treatments with 30% to 100% fungal filtrate, the increase in GR activity was more remarkable than that obtained by PAT treatments and enhanced to reach a 1.73- to 2.50-fold, as compared to control. The increase in GR activity of shoot was 1.33- to 2.0-fold of control (Figure 3b). Within all treatments of PAT (except 10 μg·mL^−1^) and fungal filtrate (except 60%), the GR activity measured in shoot were significantly higher than that measured in root.

**Figure 4:**
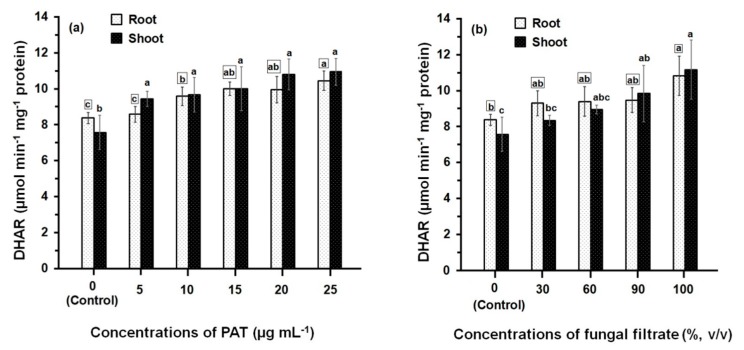
DHAR activity in root and shoot of 8-day-old maize seedlings treated with different concentrations of PAT (**a**) and fungal filtrate (**b**). Data are shown as the mean ± SD of triplicate measurements from three independent experiments. Different letters either for roots (in small squares) or shoots (without squares) at different concentrations are statistically different (LSD test, *p* ≤ 0.05).

Comparison of the DHAR activities of PAT- and fungal filtrate-treated root and shoot (Figure 4) shows that the enzyme activities in all treatments are always significantly greater than control. However, the difference in DHAR activity amongst concentrations of both PAT (Figure 4a) and fungal filtrate (Figure 4b) was relatively small. At 25 μg·mL^−1^ (the higher PAT concentration), the recorded DHAR activity in root and shoot was 10.45 ± 0.55 μmol·min^−1^·mg^−1^ and 10.94 ± 0.75 μmol·min^−1^·mg^−1^ protein, respectively; which increased 1.24- and 1.45-fold of their control treatments. Similar activities of DHAR were obtained the concentrated fungal filtrate (100%, *v/v*), where the DHAR activity in root and shoot increased 1.30- and 1.48-fold, respectively; as compared to control treatments. Results further indicated that similar DHAR activities were obtained when comparing the enzyme activities of root and shoot at different treatments of PAT and fungal filtrate.

**Figure 5:**
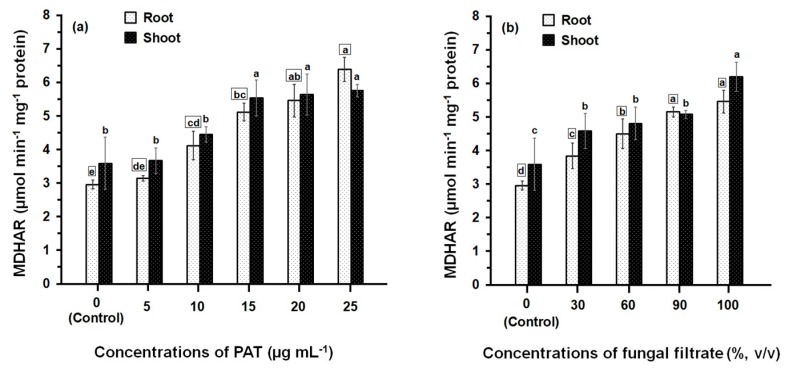
MDHAR activity in root and shoot of 8-day-old maize seedlings treated with different concentrations of PAT (**a**) and fungal filtrate (**b**). In this figure, data are shown as the mean ± SD of triplicate measurements from three independent experiments. Different letters either for roots (in small squares) or shoots (without squares) at different concentrations are statistically different (LSD test, *p* ≤ 0.05).

Treatment of maize seedlings either with PAT or fungal filtrate concentrations led to an increase in MDHAR activity (Figure 5). The greatest effects were observed with high concentrations (between 10 μg·mL^−1^ and 25 μg·mL^−1^ in the case of PAT and between 60% and 100% in the case of fungal filtrate). At 10 to 25 μg·PAT·mL^−1^, MDHAR activity in root was 1.40- to 2.16-fold higher than in control. The activity in shoot was also increased to 1.23- to 1.61-fold higher than in control (Figure 5a). Similar MDHAR activities were obtained in the root and shoot treated with 60 to 100% fungal filtrate recording 1.52- to 1.85-fold higher and 1.33- to 1.72-fold higher, respectively; than in control treatments (Figure 5b).

## 4. Discussion

It is commonly accepted that the adverse biological activity of PAT arises from its reactivity with thiol groups of biological molecules like cysteine, GSH, aminoacyl-tRNA synthetases, alcohol and lactic dehydrogenases, and muscle aldolase [13,24,25,27,34,35]. We previously demonstrated that PAT isolated from *P. vulpinum* CM1 was phytotoxic to maize seedlings [15]. The phytotoxic action of PAT including the cytomorphological modifications (in particular changes in the nature of cytoplasm and cytoplasmic organelles) and metabolic effects (alterations in the total GSH concentration) had not been previously reported. For this reason, the effect of PAT on activity of GST and antioxidant enzymes was studied as a complementary study. Using YES broth (pH 6.0) as cultivation medium and F-254 silica gel TLC plates sprayed with phenylhydrazine hydrochloride solution, the PAT production level of the *P. vulpinum* CM1 was 10.42 μg·mL^−1^. In this regard, PAT was found to be produced by different *Aspergillus* and *Penicillium* spp. including *P. claviforme* in concentrations ranging between 0.2 μg·mL^−1^ and 3127 μg·mL^−1^ using Czapek’s enriched liquid medium [36]. Additionally, PAT was produced by *P. claviforme* NRRL 1001 and NRRL 1002 using potato dextrose broth and the first strain showed a PAT producing ability ˃ 250 μg·mL^−1^ and the second strain showed a higher yield, attaining ˂ 1200 μg·mL^−1^ [37]. Production of PAT is not a generic character, though *Aspergillus* and *Penicillium* spp. are so far the most extensively represented [6,38]. In our preliminary results for mycotoxins analysis in *P. vulpinum* CM1 culture, the strain proved its positive activity for production of PAT and roquefortine C, and it showed a negative activity for production of cyclopiazonic acid and griseofulvin [15]. Moreover, our preliminary results demonstrated that roquefortine C showed a no phytotoxicity to germination of maize seeds, while PAT was identified as a responsible agent for the phytotoxic effect of the *P. vulpinum* CM1 culture filtrate. In the present study, a comparison between the effect of PAT and *P. vulpinum* culture filtrate on some enzymatic activities was investigated. Kozlovskii et al. [4] reported that the culture filtrate of *P. vulpinum* comprises several alkaloids (cyclopeptin, cyclopenin, meleagrin, and oxaline) that possess antibiotic activities and may become dangerous due to possible synergistic effects.

In this study, the effect of different concentrations of PAT and fungal filtrate on GST activity of maize was investigated. GST is a superfamily of multifunctional, dimeric enzymes that are involved in diverse aspects of biotic and abiotic stresses, especially detoxification of endo- and xenobiotics [21,39]. Their detoxification capacity is related to the ability to conjugate GSH to various targets involved in stress [21]. In maize, it was reported that there are 42 GST genes [40]. The present results demonstrated that GST activity either in the root or shoot of maize seedlings was significantly reduced following treatment with PAT concentrations varied from 5 to 25 μg·mL^−1^. The reduction percent in GST activity due to treatment with PAT was dose-related. It was in the range of 47.0% to 73.8% in root and in the range of 37.8% to 60.1% in shoot. Similar reduction percents were obtained in GST activity following treatment with fungal filtrate varied from 30 to 100% (*v/v*). Maximum reduction percent in the GST activity of both root and shoot of maize seedling was obtained upon application of the concentrated fungal filtrate (100%, *v/v*) achieving 76.0% and 61.7%, respectively. The decline in GST activity in root and shoot of maize may be explained on the basis of the mode of action toxicity of PAT and the lower values of GST activity may reflect a higher specificity of this enzyme for PAT. It is well known that PAT induces accumulation of ROS which results in increased lipid peroxidation [41]. The increase in H_2_O_2_ concentration mediates the inactivation of GST by PAT via the oxidation of their essential thiol and amino groups [35]. Moreover, Pfeiffer et al. [26] found a reduced activity of GST after PAT treatment in vitro. The second interpretation suggests that PAT impaired the biosynthesis of intermediate metabolic compound (s), thereby causing indirect inhibition of protein synthesis. The same explanation has been interpreted by Hewitt et al. [42] for the inhibitory effect of PAT on induction of nitrate reductase (either by molybdenum or nitrate). The authors further showed that PAT acts as antimetabolite inhibiting gross ribonucleic acid content and incorporation of phosphorus into the ribonucleic acid of cauliflower leaf tissues [42]. PAT was found to inhibit the eukaryotic protein synthesis and its prenylation [43,44]. A third possibility could be explained on the basis of the relationship between plant and GST activity. Plant growth and development is associated with GST enhancement [21]. GST levels have been shown to be induced by a wide variety of phytohormones such as ethylene, auxin, methyl jasmonate, salicylic acid, and abscisic acid [39,45]. Our previous study showed that PAT affects seedling growth and development of root and shoot [15] which might affect phytohormones, and in turn affect GST activity. Also, pretreatment of wheat and maize with the chloroacetanilide herbicide acetochlor induced GST activity [46].

Results of the effect of PAT and fungal filtrate concentrations on GST activity further showed that enzyme activity in roots of maize is significantly higher than that of shoots, as well as in control treatments. This result is in line with that obtained by Jablonkai and Hatzios [46] who reported higher activity of GST in root of maize than in its shoot after treatment of maize seedlings with the herbicide acetochlor. The data on the lower expression of shoot GST activity following treatment with PAT appear to correlate well with the translocation rate of PAT. The same explanation has been interpreted for the effect of the herbicide acetochlor on GST activity in shoots, in which acetochlor was found to be the most mobile [46].

The increased production of ROS induced by PAT can disrupt the redox status of cells, resulting in oxidative stress, thereby causing membrane dismantling, macromolecule deterioration, ion leakage, lipid peroxidation, and DNA cleavage [18,22]. These ROS-injuries in plants were reported to be induced by heavy metals and in order to prevent the induction of ROS-injuries, plants have developed various defense mechanisms to sustain the cellular redox state and mitigate the damage caused by oxidative stress [17]. These defense mechanisms include hindering metal entrance into plant tissues, limiting metal accumulation, chelation by organic molecules, metal binding to cell wall and sequestration in vacuoles [17]. Using the defense mechanisms, plant can transform ROS into less-toxic products. Among these defenses, high levels of AsA and GSH, as well as antioxidant enzymes such as APX, GR, DHAR, MDHAR, peroxidase, and superoxide dismutase are produced by plants [19,47]. In our previous study [15], we have previously demonstrated that various concentrations of PAT and fungal filtrate of *P. vulpinum* induced elevation of GSH content either in roots or shoots, as compared to control treatments.

In the current study, APX, GR, DHAR, and MDHAR were all detected in root and shoot of maize under the influence of fungal filtrate of *P. vulpinum* and PAT. APX is a central enzyme of AsA-GSH cycle and is a member of AsA-specific peroxidases [48]. It acts as the electron donor for the decomposition of H_2_O_2_. GR is a member of flavoenzymes and contains an essential disulfide group [49]. It is an NADPH-dependent enzyme that catalyzes the reduction of the oxidized glutathione (GSSG) to its reduced form (GSH), thus maintaining a high ratio of GSH/GSSG and playing a main role in cell metabolism [32]. DHAR is a monomeric thiol enzyme that plays an essential role in maintaining AsA in the reduced form because it catalyzes the reduction of DHA to AsA [50]. MDHAR is a FAD enzyme that catalyzes the regeneration of AsA from the monohydrate ascorbate radical using NADPH as an electron donor [51]. Our data showed that the individual treatment with fungal filtrate and PAT at all concentrations induced elevation of the antioxidative enzymatic activities of APX, GR, DHAR, and MDHAR measured either in roots and shoots, as compared with those obtained at control treatments. PAT treatment activated or stimulated a number of genes involved in the defense against oxidative stress, the metabolism of sulfur-containing amino acids, and protein degradation [52]. Reports on the elevation of antioxidant enzymes activities of selected plant species following their treatment with different types of heavy metals are available [53,54,55]. The increased levels of these metabolic intermediary compounds and of antioxidant enzymes lead to increased stress tolerance against heavy-metal-induced ROS [56]. The enhancement in the activities of these antioxidative enzymes may be due to GSH elevation and a subsequent activation in the AsA-GSH cycle. The same interpretation was explained by Gomes-Junior et al. [57] and Bashir et al. [58]. These authors reported on a relation between the activities of APX and GR and sulfur. They showed that sulfur deficiency significantly suppresses APX and GR activities and the decline in their activities is associated with depletion of GSH.

The present results showed that the enhancement of the antioxidant enzyme activities following treatment of maize seedlings either with PAT or fungal filtrate was dose-related. Similarly, Shahid et al. [19] found that the toxic effects of heavy-metal-induced ROS on plant macromolecules vary and depend on the duration of exposure, stage of plant development, concentration of heavy metals tested, intensity of plant stress, and the particular organs affected. The results obtained also indicated that the expression rates of DHAR and MDHAR in root and shoot were relatively similar. However, the activity of both APX and GR was considerably more evident in shoot than in root. These results were supported by Foyer and Halliwell [59] and Jaleel et al. [60] who reported that the bulk of APX and GR activity is found in chloroplasts of leaves, whereas root plastids exhibit a lower proportion of enzyme activity and, as indicated earlier, the activities of antioxidative enzymes in a plant organ is associated with GSH, and as the greater concentration of GSH is found in shoot than in root, hence the antioxidative enzymatic activities are more pronounced in shoots than in roots [57,58].

## 5. Conclusions

In summary, the present results have demonstrated the significant reduction of the GST activity in root and shoot tissue following application of PAT and culture filtrate of *P. vulpinum* to maize seedlings. The reduced GST activity was dose-related, assuming that PAT may interact covalently with the –SH and –NH_2_ groups of the GST, causing inactivation of the enzyme in a direct manner or impaired the biosynthesis of its metabolic intermediary compounds, causing enzymatic inactivation indirectly. NMR experiments could be performed to investigate the details of the interaction between PAT and GST. The significantly greater decrease in the GST activity in shoot compared to that in root within all treatments of PAT and fungal filtrate indicated the translocation of PAT from root to shoot. Results of analyzing the antioxidative enzymatic activities in root and shoot of PAT- and fungal filtrate-treated seedlings showed significant elevation in the activities of APX, GR, DHAR, and MDHAR when compared with control treatments. The increased antioxidative enzymatic activity was interpreted on the basis of the availability of GSH induced by PAT and the subsequent activation in the AsA-GSH cycle. This is consistent with the presumed roles of antioxidants as ROS-scavenging enzymes, suggesting a compensatory response. To test our hypothesis in more detail, GSH could be added to maize seedlings in comparison to and in combination with buthionine sulfoximine (BSO) application to reduce the endogenous GSH content and analyze the impact on APX, GR, DHAR, and MDHAR activities. A time-course experiment by measuring the impact of PAT on the different antioxidant enzyme activities and abundance levels would lead to a better understanding how the maize antioxidant system responds to PAT in a timely manner. Alternatively, PAT might also impact expression of the genes encoding antioxidant enzymes, increasing the protein abundance and needs to be analyzed in the future.

## Figures and Tables

**Table 1:** Effect of different concentrations of PAT on the GST activity of root and shoot of 8-day-old maize seedlings.

PAT Conc. (μg·mL^−1^)	Root		Shoot	
	GST (nmol^−1^·min^−1^·mg^−1^ Protein)	Reduction (%) in GST Activity	GST (nmol^−1^·min^−1^·mg^−1^ Protein)	Reduction (%) in GST Activity
0.0 (control)	272.68 ± 23.89 ^a^	0.00	113.28 ± 14.59 ^a^	0.00
5.0	144.31 ± 15.70 ^b^	47.0	70.42 ± 9.67 ^b^	37.8
10	120.71 ± 17.63 ^b,c^	55.7	64.11 ± 10.99 ^b^	43.4
15	117.28 ± 9.52 ^b,c^	57.0	59.45 ± 9.42 ^b^	47.5
20	89.75 ± 15.98 ^b,c^	67.1	56.73 ± 13.03 ^b^	50.2
25	71.38 ± 9.84 ^c^	73.8	45.19 ± 8.14 ^b^	60.1

Calculated mean is for triplicate measurements from two independent experiments ± SD, ^a b c^ different superscript letters indicate that the means in the same column are statistically different (LSD test, *p* ≤ 0.05).

**Table 2:** Effect of different concentrations of *P. vulpinum* CM1 culture filtrate on the GST activity of root and shoot of 8-day-old maize seedlings. Calculated mean is for triplicate measurements from two independent experiments ± SD.

Fungal Filtrate Conc. (%, *v/v*)	Root		Shoot	
	GST (nmol^−1^·min^−1^·mg^−1^ Protein)	Reduction (%) in GST Activity	GST (nmol^−1^·min^−1^·mg^−1^ Protein)	Reduction (%) in GST Activity
0.0 (control)	272.68 ± 23.89 ^a^	0.00	113.28 ± 14.59 ^a^	0.00
30	151.44 ± 19.03 ^b^	44.5	110.12 ± 12.09 ^a^	2.80
60	124.00 ± 15.89 ^c^	54.5	99.38 ± 12.04 ^a^	12.3
90	106.94 ± 10.26 ^c^	60.8	95.63 ± 11.56 ^a^	15.6
100	65.55 ± 4.41 ^d^	76.0	43.33 ± 2.19 ^b^	61.74

^a–d^ different superscript letters indicate that the means in the same column are statistically different (LSD test, *p* ≤ 0.05).
